# To Subscribers

**Published:** 1859-12

**Authors:** 


### To Subscribers.

We would remind our subscribers, that this is now the fourth number of the Volume, and that there is a most *startling* delinquency manifested by an examination of our books, not only in reference to the present volume, but to those which have preceded it. We would desire to be understood as making a most emphatic and earnest call upon all who are indebted for this Journal, to discharge their obligations without delay.

